# miR-16-5p Is a Novel Mediator of Venous Smooth Muscle Phenotypic Switching

**DOI:** 10.1007/s12265-022-10208-1

**Published:** 2022-05-02

**Authors:** Dengshen Zhang, Jun Shi, Guiyou Liang, Daxing Liu, Jian Zhang, Sisi Pan, Yuanfu Lu, Qin Wu, Changyang Gong, Yingqiang Guo

**Affiliations:** 1 Department of Cardiovascular Surgery, West China Hospital, Sichuan University, Chengdu, 610041 Sichuan China; 2 Department of Cardiovascular Surgery, Affiliated Hospital of Zunyi Medical University, Zunyi, 563099 Guizhou China; 3 Guizhou Medical University, Guiyang, 550025 Guizhou China; 4 Key Laboratory of Basic Pharmacology of Ministry Education, Zunyi Medical University, Zunyi, 563006 Guizhou China; 5 State Key Laboratory of Biotherapy, West China Hospital, Sichuan University, Chengdu, 610041 Sichuan China

**Keywords:** miR-16-5p, Zyxin, Vascular smooth muscle cell phenotypic switching, Intimal hyperplasia

## Abstract

Vein graft failure after coronary artery bypass grafting (CABG) is primarily caused by intimal hyperplasia, which results from the phenotypic switching of venous smooth muscle cells (SMCs). This study investigates the role and underlying mechanism of miR-16-5p in the phenotypic switching of venous SMCs. In rats, neointimal thickness and area increased over time within 28 days after CABG, as did the time-dependent miR-16-5p downregulation and SMC phenotypic switching. Platelet-derived growth factor-BB-induced miR-16-5p downregulation in HSVSMCs was accompanied by and substantially linked with alterations in phenotypic switching indicators. Furthermore, miR-16-5p overexpression increased SMCs differentiation marker expression while suppressing HSVSMCs proliferation and migration and drastically inhibiting neointimal development in vein grafts. The miR-16-5p inhibited zyxin expression, which was necessary for HSVSMCs phenotypic switching. The miR-16-5p/zyxin axis is a novel, potentially therapeutic target for preventing and treating venous graft intimal hyperplasia.

**Graphical abstract:**

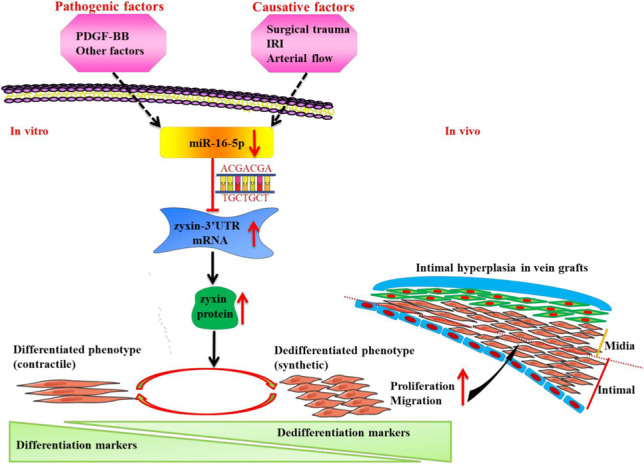

**Supplementary Information:**

The online version contains supplementary material available at 10.1007/s12265-022-10208-1.

## Introduction

In 2017, 126 million people worldwide were affected by coronary artery disease (CAD), accounting for 1.72% of the world’s population [1, 2]. The saphenous vein is the most commonly used autograft vessel in coronary artery bypass grafting (CABG), the primary surgical treatment for CAD, and the saphenous vein represents the most common autograft vessel used in CABG [3]. However, nearly half of the vein grafts fail within 10 years, mainly resulting from intimal hyperplasia [4–6]. Many studies have demonstrated that smooth muscle cells (SMCs) with high plasticity can switch from a differentiated phenotype (contractile type) to a dedifferentiated phenotype (proliferative migratory phenotype) when triggered by suitable factors, a process known as SMC phenotypic transformation. The phenotypic switching of vascular smooth muscle cells (SMCs), which is associated with increased cell proliferation and migration, promotes neointimal development in vein grafts and is a therapeutic target for preventing intimal hyperplasia [7–9]. Many miRNAs have been shown to regulate the phenotypic switching of SMCs [10–12]. The miR-21, for example, is upregulated in mouse, pig, and human vein graft models. By reducing SMC proliferation in the neointimal layer, miR-21 knockdown inhibits intimal hyperplasia in mouse vein grafts [13]. Endogenous miR-22 silencing, on the other hand, promotes SMC proliferation by suppressing the expression of methyl-CpG binding protein 2 and histone deacetylase 4 [14]. Moreover, dedifferentiation of SMCs contributes to the development of proliferative vascular disorders such as vein graft restenosis, arterial injury stenosis, and hypertensive vascular remodeling [15]. The miR-145 and miR-663 are prevalent in vascular SMCs, but they are downregulated in response to platelet-derived growth factor-BB (PDGF-BB). SMC proliferation and vascular neointimal formation are inhibited by restoring miR-145 and miR-663 expression [16, 17]. As a result, a greater understanding of how miRNAs influence phenotypic switching of venous SMCs may lead to more therapeutic targets for combating intimal hyperplasia in vein grafts.

According to research studies, miR-16-5p plays a role in regulating cell differentiation, proliferation, and migration. For instance, Liu et al. discovered that miR-16-5p promotes bone marrow mesenchymal stem cell (BMSC) differentiation into cardiac myoid cells [18]. Huang et al. demonstrated that miR-16-5p expression is considerably reduced in rat hypertrophic myocardium and its restoration effectively promotes cardiac contractile function recovery [19]. Furthermore, the loss of miR-16-5p is linked to the development of human malignancies such as chronic lymphocytic leukemia, chordate tumors, and lung cancer. In addition, miR-16-5p overexpression in these tumor cells may regulate cell phenotypes and limit cell proliferation, migration, and invasion [20–22]. Since intimal hyperplasia is associated with SMCs differentiation that facilitates cell proliferation and migration [7–9], it can be hypothesized that miR-16-5p might play a role in developing intimal hyperplasia by regulating SMCs phenotypic switching.

## Methods

### Ethical Approval

Human saphenous vein segments were acquired from patients who underwent CABG procedures at West China Hospital, Sichuan University (Chengdu, China) between January 2018 and March 2020. This study was approved by The Clinical Trials and Biomedical Ethics Committee of West China Hospital, Sichuan University (No. 2019804). The animal study was approved by the Animal Research Committee of West China Hospital.

### Cell Culture and PDGF-BB Treatment

As previously described, HSVSMCs were isolated from saphenous vein segments obtained from patients undergoing CABG surgeries [23, 24]. For cell culture, SMC medium (Siencell, Carlsbad, CA, USA) supplemented with 15% fetal bovine serum was used (FBS; Gibico, Thermo Fisher Scientific, Waltham, MA, USA). HSVSMCs was verified by immunofluorescence staining for smooth muscle-specific α-actin (SMα-actin) and smooth muscle 22α (SM22α). All experiments employed HSVSMCs at passages 3–5. For PDGF-BB treatment, HSVSMCs were cultured to 70–80% confluence and then serum-starved for 24 h before being incubated with human PDGF-BB for 24 h again (R&D Systems, Minneapolis, MN, USA) in SMC medium containing 0.5% FBS, as previously described [16, 17]. 293T cells were cultured in Dulbecco’s modified Eagle’s medium (Gibco) supplemented with 10% FBS.

### Vein Graft Model

The vein graft model was established in rats utilizing the previously described end-to-end anastomotic approach [25, 26]. In brief, male Sprague–Dawley rats (250 ± 30 g) were anesthetized with 2% isoflurane (Lunan Beit Pharmaceutical Co., Ltd., China) at an oxygen flow rate of 1 L/min using an isoflurane vaporizer (Matrx VIP 3000, Midmark, USA) [27]. The subsequent procedure was carried out with a surgical microsystem (Leica M220-F12, Germany). Blunt dissection separated the right external jugular vein and common carotid artery from the surrounding tissues. The common carotid artery was then ligated using 8.0 nylon sutures at the midpoint and divided. Each anastomosis was sutured with 8 to 10 stitches using 8.0 nylon sutures after the right external jugular vein (5–15 mm in length) was dissected and transplanted into the right common carotid artery. Intraperitoneal injection of sodium pentobarbital (> 0.2 mL/100 g; Wuhan Yitai Tech Co., Ltd., China) was used at 7, 14, or 28 days after surgery. The vein grafts were collected for further study. As a control, the left jugular vein was used.

### Lentivirus Production

This investigation employed the lentiviral vector pHBLV-U6-MCS-CMV-ZsGreen-PGK-puro (Hanbio Biotechnology, China). The vector carrying miR-16-5p (Lv-miR-16-5p), antisense-miR-16-5p (anti-miR-16-5p), or corresponding negative control (Lv-NC or LV-anti-NC) was constructed by inserting the miR-16-1 precursor, the reverse complementary sequence of miR-16-5p, or corresponding negative control sequence into the vector. By inserting the reverse complementary sequence of zyxin (5′-GTGTTACAAGTGTGAGGAC-3′) or the corresponding negative control sequence into the vector, the vector carrying small hairpin RNA against zyxin (shRNA-zyxin) [28] or the corresponding negative control sequence (shRNA-NC) was constructed. Lentiviruses were packaged in 293T cells (Academy of Sciences cell repository, China) and purified by ultracentrifugation, as previously described [29]. The infections were carried out using lentivirus at a concentration of 3.0 × 108 T.U./mL. The miR-16-1 precursor (MI0000070, miRBase) and anti-miR-16-5p (MI0000069, miRBase) sequences were as follows:

5′-GTCAGCAGTGC CTTAGCAGCACGTAAATATTGGCGTTAAGATTCTAAAATTATCTCCAGTATTAACTGTGCTGCTGAAGTAAGGTTGAC-3′ and 5′-ACAGGATCCCGCCAATATTTACGTGCTGCTATATACCGCCAATATTTACGTG CTGCTAACATCCGCCAATATTTACGTGCTGCTATCTTCACGCCAATATTTACGTGCTGCTATTTTTTGAATTCACA-3′ [28].

### Transduction and Transfection

The delivery of Lv-miR-16-5p or Lv-NC into the vein grafts was performed using N, O-carboxymethyl chitosan (NOCC)/aldehyde hyaluronic acid (AHA) hydrogel as previously described [30, 31]. NOCC (20 mg/mL) and AHA (30 mg/mL) were dissolved in phosphate-buffered saline (PBS) for 2 h at 37 °C and then stored at 4 °C for 7 days. The solutions were filtered (0.22-m pore size) and combined in a 1:1 ratio to make the hydrogel. Moreover, to optimize lentiviral vector delivery into vein grafts, the exterior membrane of the vein graft was evenly coated with 100 μL NOCC/AHA containing 0, 5, 10, or 20 μL (3108 TU/mL) of Lv-ZsGreen. In addition, immunofluorescence analysis was performed (Supplementary Fig. S1), and a 100 L NOCC/AHA hydrogel containing 20 L lentiviral vectors (3 × 108 T.U./mL) was used in the following experiments to measure transduction effectiveness. At 28 days after transduction, the vein grafts were harvested.

The lentiviral vectors were transduced into HSVSMCs at a concentration of 3.0 × 108 TU/mL. For miR-16-5p/zyxin co-overexpression, HSVSMCs were co-transfected with Lv-miR-16-5p and zyxin-overexpressing plasmid pcDNA3.1-EF1a-mcs-3flag -CMV-EGFP (Hanbio Biotechnology, China) using 0.5 μg LipoFiterTM transfection reagent (Hanbio Biotechnology) per 105 HSVSMCs following the manufacturer’s instructions.

### Quantitative Real-Time PCR

Total RNA was extracted from rat grafted veins or HSVSMCs using Trizol (Thermo Fisher Scientific), followed by cDNA synthesis using the PrimeScript™ RT reagent kit (Takara, Japan) manufacturer’s instructions. An SYBR green kit (Takara, Japan) and specific primers for miR-16-5p, SMα-actin, SM22α, osteopontin (OPN), proliferating cell nuclear antigen (PCNA), and zyxin were used for PCR (Supplementary Table S1). U6 and GAPDH were used as internal references for miR-16-5p and other genes, respectively. The 2^−ΔΔCt^ technique was used to calculate gene expression.

### Western Blot Analysis

RIPA buffer containing 1% phenylmethylsulfonyl fluoride and 1% protease inhibitor extracted total proteins from rat grafted veins or HSVSMCs (Solarbio, China). Protein concentrations were determined using a BCA assay kit (Solarbio). Proteins (10–30 g/lane) were separated by sodium dodecyl sulfate-polyacrylamide gel electrophoresis (8% or 10% gels) and transferred to PDGF membranes in equal proportions. Following blocking with 10% non-fat milk, the membranes were incubated with anti-zyxin (1:1,000; Abcam, Cambridge, UK), anti-SMα-actin (1:2,000, Abcam), anti-SM22α (1:1,000, Abcam), anti-OPN (1:1,000, Abcam), anti-PCNA (1:1,000, Abcam), or anti-GAPDH (1:5,000, Abcam) antibodies, followed by incubation with HRP-conjugated AffiniPure goat anti-rabbit IgG (H+L) (1:5,000; Proteintech, Wuhan, Hubei, China). The ChemiDoc MP imaging system captured images of immunoreactive bands (Bio-Rad, China). Image Lab software was used to calculate the band densities (Bio-Rad).

### Dual-Luciferase Reporter Assay

Wild-type or mutant zyxin 3′-untranslated region (UTR) fragments were cloned into a pSI-Check2 luciferase reporter (Hanbio Biotechnology) and co-transfected into 293T cells with miR-16-5p mimics or negative control. Luciferase activity was evaluated 48 h after transfection.

### Cell Proliferation Assay

The MTT assay and EdU staining were used to measure HSVSMC proliferation. In 96-well plates, HSVSMCs were seeded at a density of 5.0 × 104 cells/mL for the MTT assay. After transfection, cells were serum-starved for 24 h before being incubated in media containing 0.5% FBS and 20 ng/mL PDGF-BB for 24 h. The media was then supplemented with MTT solution (Solarbio) (1:10), and the cells were cultured for 4 h before adding 110 μL formazan solution to each well. After a 10-min incubation period, the absorbance was measured at 490 nm.

EdU staining was carried out according to the manufacturer’s instructions using an EdU assay kit (Solarbio). In 12-well plates, HSVSMCs were seeded at a density of 5.0 × 104 cells/mL. The cells were treated in the manner described above. Cell proliferation was quantified as the percentage of EdU-positive cells in DAPI-positive cells.

### Cell Migration Assay

Cell migration assays were performed using Transwell chambers with 8-μm pores (Corning, U.S.A.) [32]. HSVSMCs were transfected as previously described and then seeded into upper chambers containing media supplemented with 0.5% FBS and 20 ng/mL PDGF-BB. The lower chambers were filled with medium containing 10% FBS. The migrating cells were fixed with methanol and stained with crystal violet after 24 h. The migrating cells were counted using ImageJ software (NIH, Bethesda, MD, USA).

### Hematoxylin and eosin staining and immunohistochemical staining

The dissected vein grafts were fixed in 4% paraformaldehyde overnight at 4 °C before being embedded in paraffin. The sections (3.5-μm thick) were stained with hematoxylin and eosin and examined according to previously reported techniques [25, 27]. The immunohistochemical staining was performed using a conventional methodology and the manufacturer's instructions with the primary antibody (Abcam) against zyxin (1:100), OPN (1:100), or PCNA (1:100).

### Statistical Analysis

SPSS software (version 22.0; IBM, Armonk, NY, USA) was used to analyze the data, expressed as the mean ± standard deviation. Statistical significance was assessed using ANOVA followed by an unpaired student's *t* test. P < 0.05 was regarded as statistically significant.

## Results

### MiR-16-5p Downregulation Accompanies SMCs Phenotypic Switching and Intimal Hyperplasia in the Vein Grafts

**Fig. 1:**
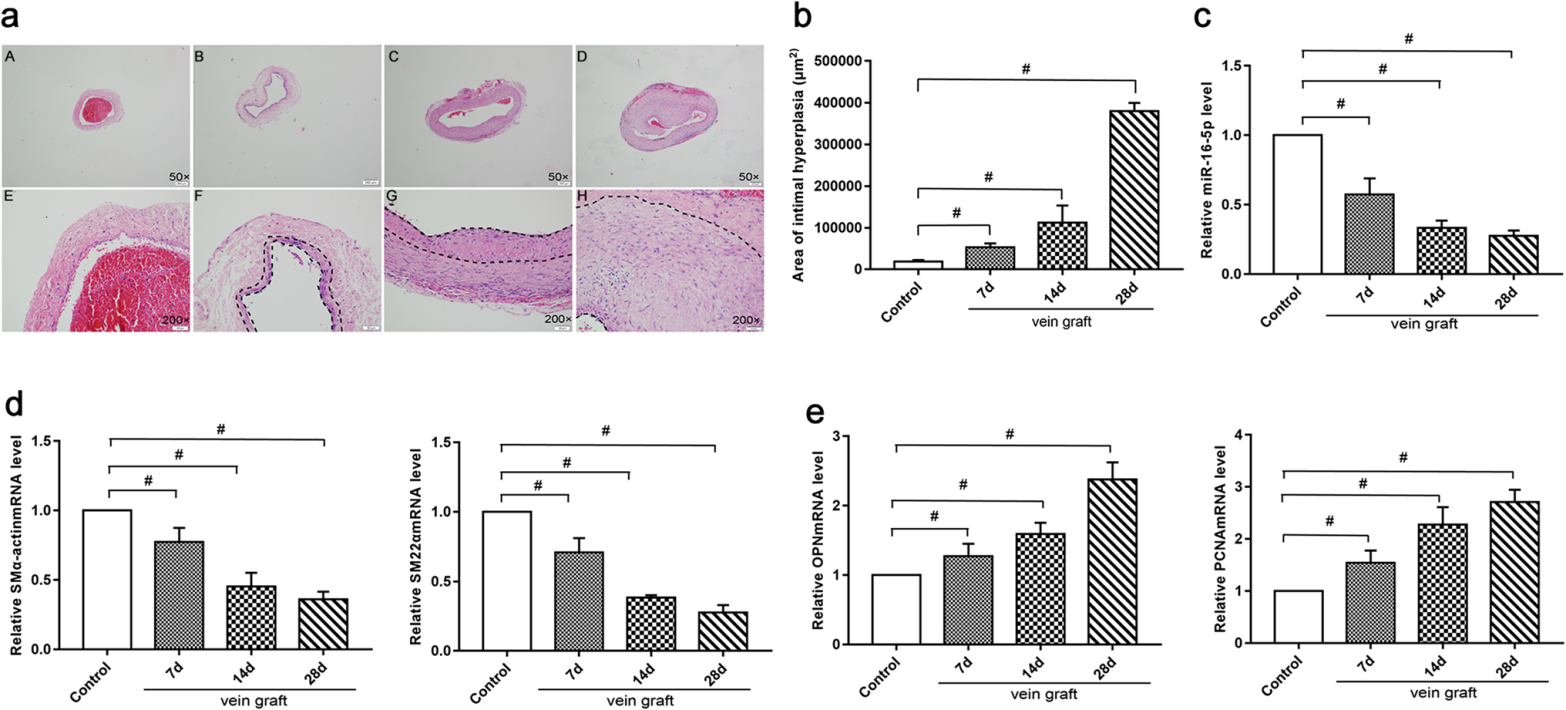
miR-16-5p level was reduced in the vein grafts in rats. A coronary artery bypass grafting (CABG) model was established in male Sprague–Dawley rats by autologous transplantation of the right external jugular vein (5–15 mm in length) into the common carotid artery. Rats were euthanized at 7, 14, or 28 days after surgery, and the vein grafts were collected. **a** (A–H) Hematoxylin and eosin (HE) staining was performed to measure the neointimal area (**c**). The dotted line indicates the neointimal area. **d–e** Quantitative real-time PCR (qRT-PCR) was conducted to measure miR-16-5p expression and mRNA levels of smooth muscle-specific α-actin (SMα-actin), smooth muscle 22α (SM22α), osteopontin (OPN), and proliferating cellular nuclear antigen (PCNA) in the vein grafts. GAPDH was used as an internal reference. U6 was used as an internal reference. The left jugular vein was used as a control. Data are expressed as the mean ± standard deviation (SD), ^#^ *P* < 0.05

A vein graft model in rats was used to study the role of miR-16-5p in phenotypic switching of venous SMCs and intimal hyperplasia. Hematoxylin and eosin (HE) staining revealed that, compared to the contralateral jugular vein, the area of the vein grafts increased significantly over time within 28 days following surgery (Fig. 1a, b), indicating that intimal hyperplasia occurs in the vein grafts. Then, in the vein grafts, we examined the level of miR-16-5p and the mRNA expression of SMC phenotypic markers. The quantitative real-time PCR (qRT-PCR) results showed that the miR-16-5p level in the vein graft was significantly reduced over time compared to that in the contralateral jugular vein (Fig. 1c), its expression had no statistical significance in contralateral jugular veins (Supplementary Fig. S2), and the mRNA levels of SMα-actin and SM22α were significantly decreased (Fig. 1d). In contrast, the mRNA levels of OPN and PCNA were increased considerably (Fig. 1e). These findings imply that downregulation of miR-16-5p has been involved in SMC phenotypic switching and intimal hyperplasia in vein grafts.

### MiR-16-5p Is Downregulated During Phenotypic Switching of HSVSMCs

**Fig. 2:**
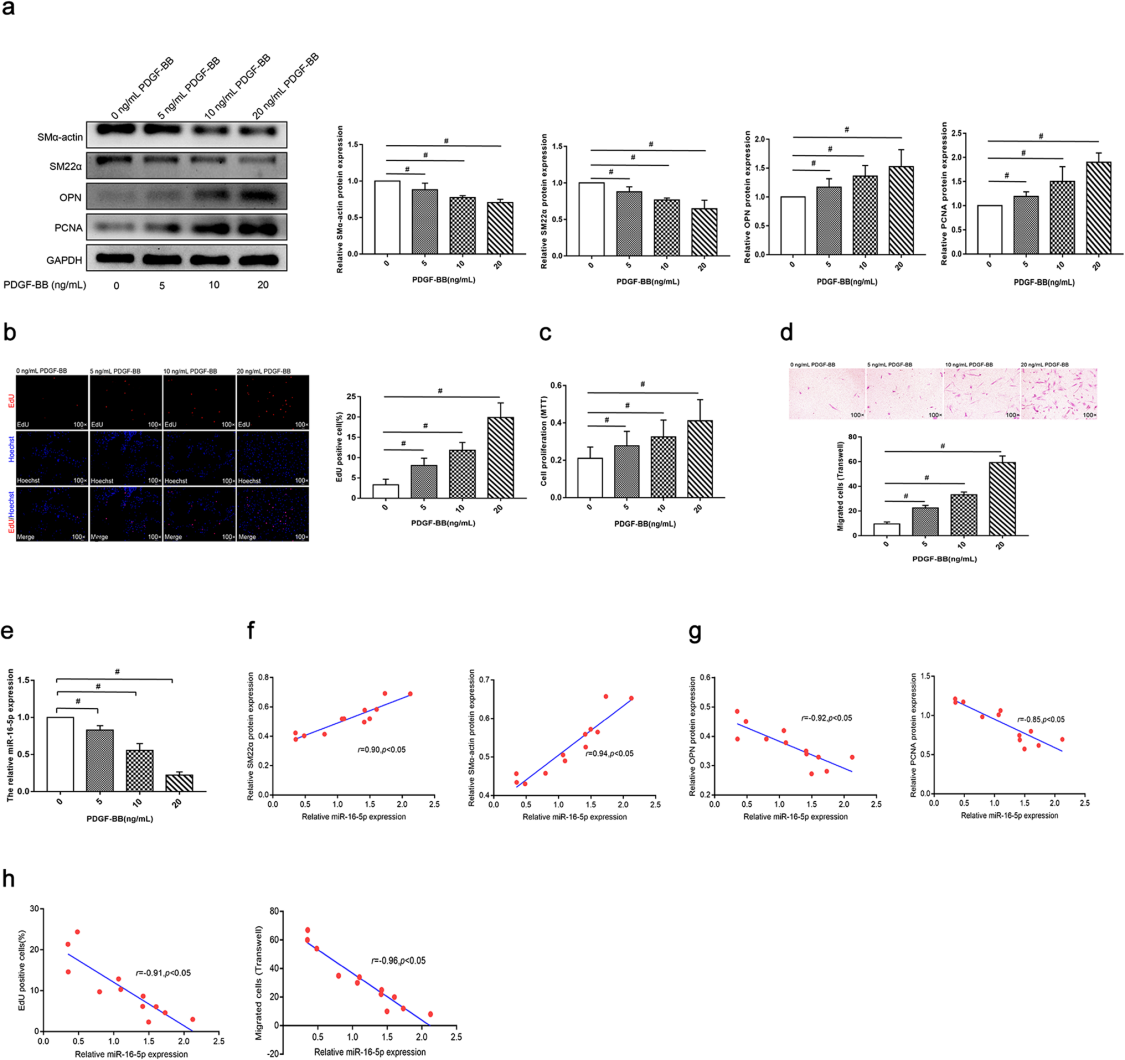
miR-16-5p level was decreased during phenotype switching of human saphenous vein smooth muscle cells (HSVSMCs) in vitro HSVSMCs were isolated from the segments of saphenous veins obtained from patients during CABG surgeries. HSVSMCs at passages 3–5 were used in all experiments. HSVSMCs were cultured to 70–80% confluence and then starved in serum-free DMEM (starvation medium I) for 24 h, followed by 24-h incubation with different concentrations of human PDGF-BB (0, 5, 10, or 20 ng/mL) in DMEM containing 0.5% fetal bovine serum (starvation medium II). **a** Western blot analysis was performed to measure protein levels of SMα-actin, SM22α, OPN, and PCNA in HSVSMCs. GAPDH was used as an internal reference. **b**–**d** EdU, MTT, and Transwell assays were conducted to examine cell proliferation and migration of HSVSMCs. **e** qRT-PCR was performed to determine miR-16-5p expression in HSVSMCs. U6 was used as an internal reference. Data are expressed as the mean ± SD. ^*#*^ *P* < 0.05; *n* = 3. **f**–**h** Spearman analysis was carried out to evaluate the correlations of miR-16-5p expression with phenotypic marker expression, Edu-positive cell percentage, and migrating cell counts

To study the involvement of miR-16-5p in the phenotypic switching of venous SMCs, we isolated HSVSMCs from patients’ saphenous veins. We measured the expression of phenotypic markers and cell proliferation and migration of HSVSMCs in response to PDGF-BB stimulation. The isolated HSVSMCs exhibited various morphologies, including spindle, linear, triangle, and stellate shapes. Immunofluorescence staining revealed that SMα-actin and SM22α were expressed strongly in more than 90% of the isolated cells (Supplementary Fig. S3), indicating that HSVSMCs were substantially enriched in the isolated cells and can be employed in the following study. Moreover, PDGF-BB treatment significantly reduced SMα-actin and SM22α protein expression while simultaneously increasing OPN and PCNA protein expression in HSVSMCs in a dose-dependent manner (Fig. 2a), which was accompanied by considerably increased cell proliferation and migration (Fig. 2b–d). These findings indicate that we successfully developed an in vitro model of PDGF-BB-induced phenotypic switching of HSVSMCs. Notably, PDGF-BB treatment dramatically reduced miR-16-5p expression in HSVSMCs in a dose-dependent manner compared to control (Fig. 2e). The Spearman correlation analysis revealed that miR-16-5p expression was substantially correlated with phenotypic switching indicators (Fig. 2f–h). These results suggest that miR-16-5p downregulation plays a role in HSVSMC phenotypic switching.

### Downregulation of miR-16-5p Mediates Phenotypic Switching of HSVSMCs

**Fig. 3:**
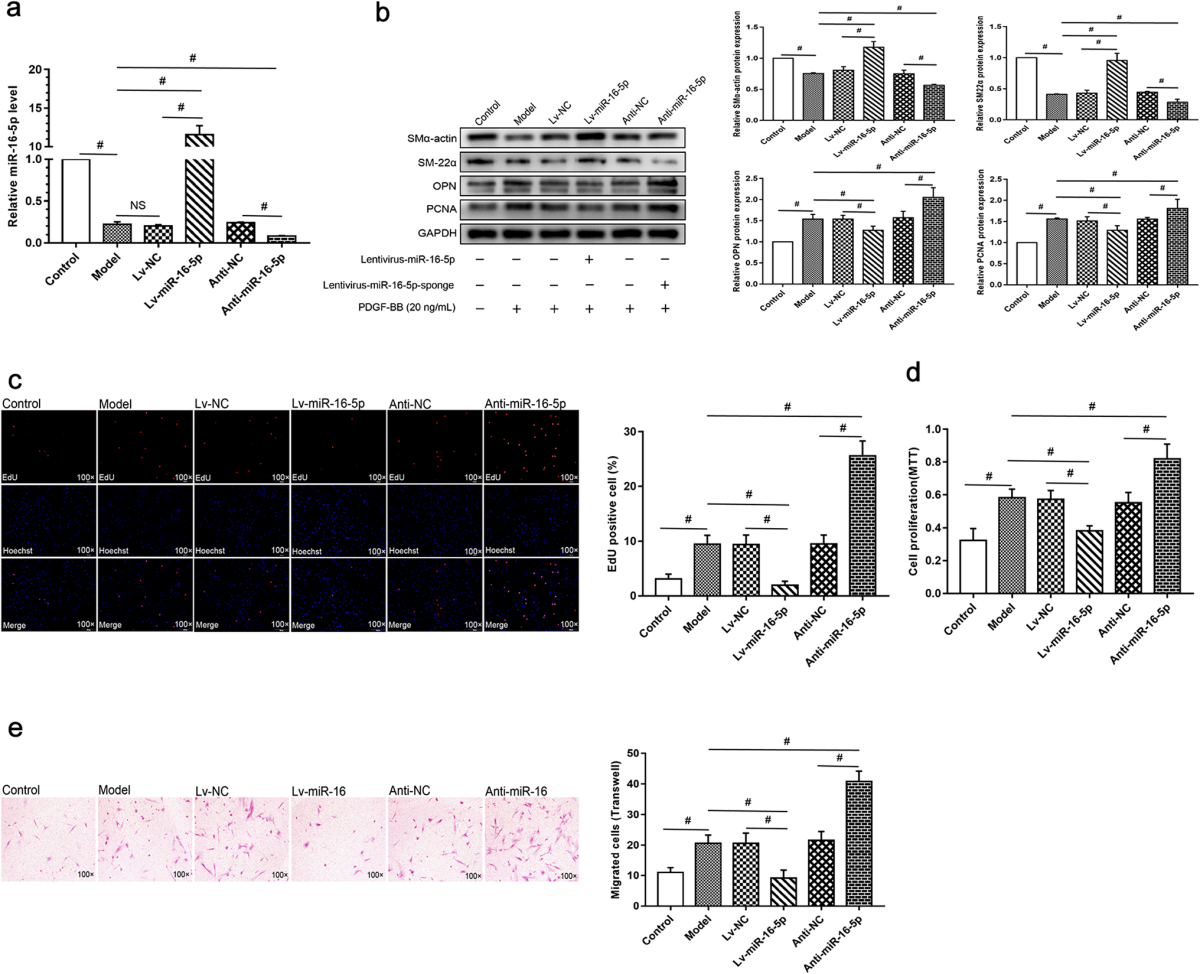
miR-16-5p regulated phenotype switching of HSVSMCs in vitro. HSVSMCs were transduced with Lv-miR-16-5p, antisense (anti)-miR-16-5p, or corresponding negative controls at a multiplicity of cellular infection at 75, as indicated. **a** qRT-PCR was performed to measure miR-16-5p expression at 48 h after transduction. **b–e** After transduction, HSVSMCs were cultured in starvation medium I for 24 h, followed by 24-h incubation with 20 ng/mL PDGF-BB in starvation medium II. Western blot analysis (**b**), EdU (**c**), MTT (**d**), and Transwell (**e**) assays were performed to examine protein expression of phenotypic markers, cell proliferation, and migration. Data are expressed as the mean ± SD, ^#^ *P* < 0.05; *n* = 3

We used gain-and loss-of-function assays in HSVSMCs exposed to PDGF-BB to see if downregulation of miR-16-5p mediates SMC phenotypic switching. After transduction with appropriate lentiviral vectors, qRT-PCR results verified the overexpression or knockdown of miR-16-5p (Fig. 3a). In the presence of PDGF-BB, we found that compared with the corresponding negative control, miR-16-5p overexpression dramatically increased protein expression of SMα-actin and SM22α while inhibiting protein expression of OPN and PCNA, whereas miR-16-5p silencing had the reverse effect (Fig. 3b). EdU, M.T.T., and cell migration assays revealed that miR-16-5p overexpression effectively reversed PDGF-BB-induced cell proliferation and migration, whereas miR-16-5p silencing further increased cell proliferation and migration of HSVSMCs (Fig. 3c–e). These findings imply that miR-16-5p downregulation causes phenotypic switching in venous SMCs.

### MiR-16-5p Modulates HSVSMCs Phenotypic Switching by Targeting Zyxin

**Fig. 4:**
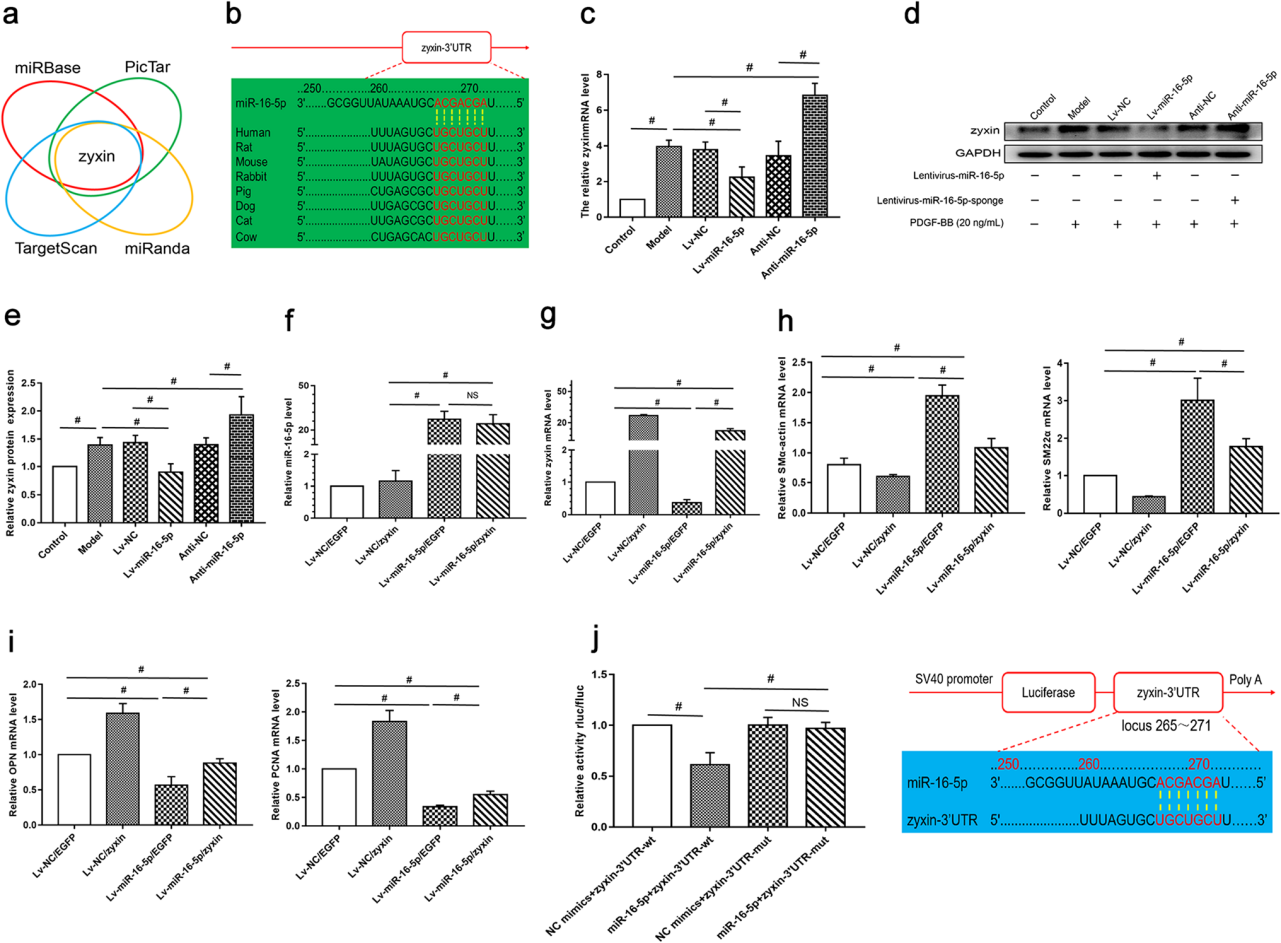
miR-16-5p targeted zyxin in phenotype switching of HSVSMCs. **a** Prediction of putative target genes of miR-16-5p using different bioinformatics databases. **b** The binding of miR-16-5p with the 3′-untranslated regions (3′-UTR) of zyxin from different species. Red letters indicate the potential binding sites. **c**–**e** HSVSMCs were transduced with Lv-miR-16-5p, anti-miR-16-5p, or corresponding negative controls, as indicated, followed by PDGF-BB (20 ng/mL) treatment as mentioned above. **c**–**e** qRT-PCR and Western blotting were performed to measure the mRNA and protein levels of zyxin in HSVSMCs. GAPDH was used as an internal reference. Control: untreated HSVSMCs; model: HSVSMCs treated with PDGF-BB without transduction. **f–i** HSVSMCs were co-transfected with Lv-miR-16-5p and zyxin-overexpressing vector or corresponding negative controls, as indicated, followed by PDGF-BB (20 ng/mL) treatment as mentioned above. qRT-PCR was performed to measure the expression of miR-16-5p (**f**), zyxin (**g**), and phenotypic markers (**h**–**i**) in HSVSMCs. Data are expressed as the mean ± SD. ^#^ *P* < 0.05. *n* = 3. **j** The 293T cells were co-transfected with miR-16-5p mimics + vectors overexpressing wildtype zyxin-3′-UTR, miR-16-5p mimics + vectors overexpressing mutant zyxin-3′-UTR, or corresponding negative controls, as indicated. The luciferase activity was measured at 48 h after transfection. Data are expressed as the mean ± SD, ^#^ *P* < 0.05; *n* = 3; *ns* non-significant, *wt* wildtype, *mut* mutant, *UTR* untranslated region. A schematic diagram of miR-16-5p-zyxin-3′-UTR binding is shown. The red letters indicate the potential binding sites

To examine the mechanism underlying the role of miR-16-5p in SMCs’ phenotypic switching, we performed bioinformatics analysis to predict the potential target of miR-16-5p. Various bioinformatics tools consistently identified zyxin as a potential target of miR-16-5p, as shown in Fig. 4a. Moreover, Fig. 4b reveals that miR-16-5p can bind to the 3′-UTR of zyxin mRNA, which is highly conserved across species. Then, we examined whether miR-16-5p influences zyxin expression in HSVSMCs. qPCR and Western blot analysis revealed that miR-16-5p overexpression significantly suppressed zyxin mRNA and protein expression, whereas miR-16-5p silencing remarkably enhanced zyxin mRNA and protein expression in HSVSMCs exposed to PDGF-BB (Fig. 4c–e). These findings imply that miR-16-5p suppresses zyxin expression in HSVSMCs.

To investigate whether miR-16-5p influences HSVSMCs phenotypic switching via zyxin, we co-overexpressed miR-16-5p and zyxin in HSVSMCs. The qRT-PCR results confirmed the restoration of zyxin expression following co-transfection (Fig. 4 f and g). In addition, we discovered that zyxin overexpression alone enhanced HSVSMC phenotypic switching, but miR-16-5p overexpression alone reduced HSVSMC phenotypic switching, as evidenced by altered mRNA expression of phenotypic markers. Importantly, co-overexpression of miR-16-5p and zyxin partially but significantly reversed the alterations in phenotypic marker expression caused by miR-16-5p overexpression alone (Fig. 4 h and i), implying that miR-16-5p affects HSVSMC phenotypic switching somewhat through zyxin.

Furthermore, the luciferase research results revealed that compared to control microRNA, miR-16-5p mimics significantly decreased the luciferase activity of the reporter vector expressing wild-type zyxin-3′-UTR, not the reporter vector expressing mutant zyxin-3′-UTR (Fig. 4j). These findings show that miR-16-5p regulates HSVSMC phenotypic switching via inhibiting zyxin expression by binding to the zyxin-3′-UTR.

### Overexpression of miR-16-5p Attenuates Neointimal Formation in the Vein Grafts

**Fig. 5:**
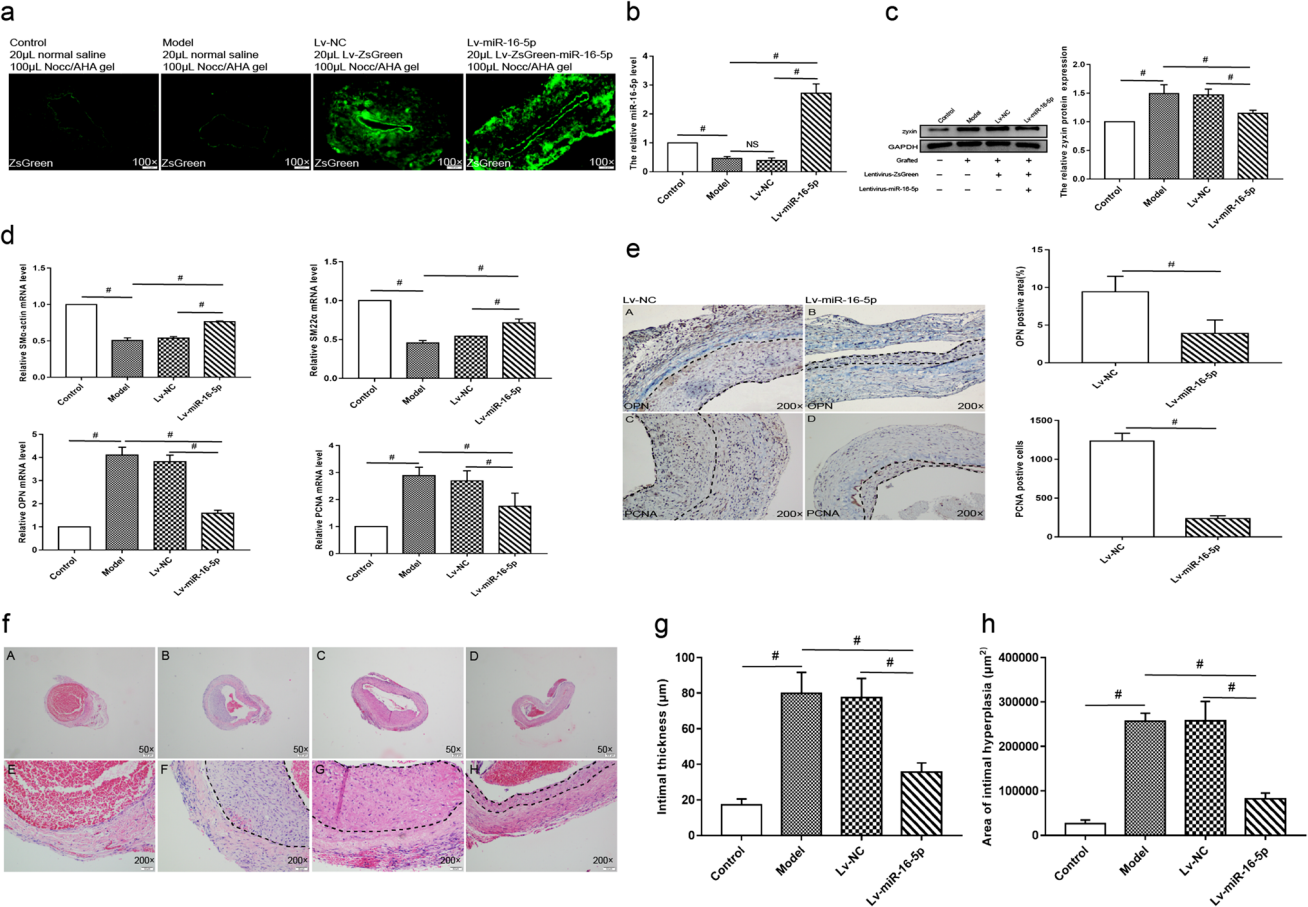
Restoration of miR-16-5p expression attenuated neointimal formation of the vein grafts in rats. The surface of the rat vein grafts was evenly applied 100 μL N, O-carboxymethyl chitosan/aldehyde hyaluronic acid (NOCC/AHA) hydrogel containing 20 μL normal saline (model), 20 μL Lv-NC (3 × 108 T.U./mL), or 20 μL Lv-miR-16-5p (3 × 108 T.U./mL) immediately after CABG. The left jugular vein applied 100 μL hydrogel containing 20 μL normal saline was used as a control. The vein grafts were harvested at 28 days after surgery. **a** Green fluorescence of the lentiviral vectors was observed under a fluorescence microscope. **b** qRT-PCR was performed to measure miR-16-5p expression in the vein grafts. U6 was used as an internal reference. **c** Western blot analysis was performed to measure protein levels of zyxin in in the vein grafts. **d** qRT-PCR was performed to measure mRNA levels of SMα-actin, SM22α, OPN, and PCNA in the vein grafts. GAPDH was used as an internal reference. **e** Immunohistochemical (IHC) staining was conducted to detect OPN and PCNA expression in the vein grafts. PCNA-positive cells were counted. The integral optical density of OPN-positive area (%) was measured. **f**–**h** HE staining was carried out to measure the neointimal thickness (**g**) and area (**h**) of the vein grafts. The dotted line indicates the neointimal area. Data are expressed as the mean ± SD, ^#^ *P* < 0.05

We overexpressed miR-16-5p in the exterior membrane of vein grafts to study its function in neointimal development. As shown in Fig. 5a, miR-16-5p was successfully administered into the grafted veins via NOCC/AHA hydrogel, as shown in Fig. 5a. qRT-PCR confirmed that Lv-miR-16-5p transduction increased miR-16-5p expression by 7.1-fold in vein grafts (Fig 5b). In grafted veins, zyxin protein expression is significantly elevated (Fig. 5c). Overexpression of miR-16-5p along with suppressed zyxin expression (Fig. 5c) increased SMα-actin and SM22α transcription, and attenuated OPN and PCNA transcription (Fig. 5d). Immunohistochemical staining confirmed the reduction in OPN and PCNA protein expression in miR-16-5p-overexpressing vein grafts (Fig. 5e), implying that miR-16-5p overexpression prevents SMC phenotypic switching. Furthermore, HE staining revealed that neointimal thickness and area, which were significantly increased in un-transduced or Lv-NC-transduced vein grafts, were substantially decreased in miR-16-5p-overexpressing veins (Fig. 5f–g). These findings show that miR-16-5p inhibits neointimal formation by reducing SMC phenotypic switching.

### Zyxin Is Required for Phenotypic Switching of HSVSMCs and Intimal Hyperplasia in the Vein Grafts

**Fig. 6:**
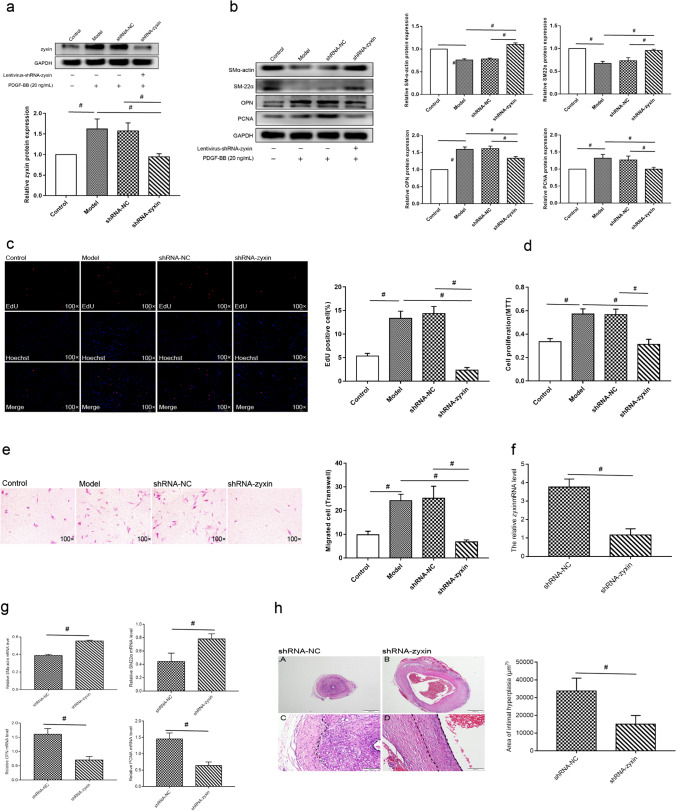
Zyxin was required for phenotypic switching of HSVSMCs in vitro and intimal hyperplasia in the vein grafts HSVSMCs were transduced with lentiviral vectors encoding small hairpin RNA against zyxin (shRNA-zyxin) and corresponding negative control (shRNA-NC), followed by PDGF-BB (20 ng/mL) treatment in vitro as described above. **a**, **b** Western blot analysis was performed to measure protein levels of zyxin (**a**) and phenotypic markers (**b**) in HSVSMCs. GAPDH was used as an internal reference. Grafted vein was transduced with lentiviral vectors encoding small hairpin RNA against zyxin (shRNA-zyxin) and corresponding negative control (shRNA-NC) in vivo. **f** qPCR was conducted to detect the expression of SMα-actin, SM22α, OPN, and PCNA. **h** HE staining was carried out to measure the neointimal area of the vein grafts. Data are expressed as the mean ± SD, ^#^ *P* < 0.05; control: untreated cells. Model: cells treated with PDGF-BB without transduction. **c–e** EdU (**c**) and MTT (**d**), and Transwell (**e**) assays were conducted to examine cell proliferation and migration of HSVSMCs. Data are expressed as the mean ± SD, ^#^ *P* < 0.05

Based on these findings, we hypothesized that during PDGF-BB-induced HSVSMC phenotypic switching, downregulation of miR-16-5p reduces the suppression of zyxin expression, promoting HSVSMC phenotypic switching. As a result, we wanted to investigate if zyxin is essential for HSVSMC phenotypic switching. Western blot analysis showed that PDGF-BB stimulation dramatically increased zyxin protein expression in HSVSMCs compared to control (Fig. 6a), accompanied by considerable downregulation of SMα-actin and SM22α and upregulation of OPN and PCNA (Fig. 6b). Notably, zyxin knockdown prevented PDGF-BB-induced changes in the expression of phenotypic markers (Fig. 6b). Furthermore, EdU, M.T.T., and Transwell tests revealed that zyxin knockdown completely inhibited PDGF-BB-induced cell proliferation and migration of HSVSMCs (Fig. 6c–e). Then, we confirmed that, as compared to the corresponding negative control, zyxin knockdown dramatically increased the expression of SMα-actin and SM22α while reducing the expression of OPN and PCNA (Fig. 6f–g) and inhibited the neointimal area in the grated vein (Fig. 6f–h). These findings imply that zyxin is essential for HSVSMC phenotypic switching and intimal hyperplasia in the vein graft.

## Discussion

The phenotypic switching of SMCs from a contractile to a highly proliferative synthetic state promotes intimal hyperplasia, which is essential in vein graft restenosis [7–9]. The miR-16-5p was shown to be downregulated during intimal hyperplasia in rat vein grafts as well as in HSVSMCs exposed to the dedifferentiation inducer PDGF-BB. Overexpression of miR-16-5p attenuated neointimal development in vein grafts and suppressed HSVSMC phenotypic switching in vitro. Mechanistically, miR-16-5p inhibited zyxin expression via binding to the 3′-UTR of the zyxin gene. Furthermore, restoring zyxin expression reversed the effect of miR-16-5p overexpression on HSVSMC phenotypic switching. Together, our results imply that miR-16-5p downregulation mediates phenotypic switching of venous SMCs by relieving the suppression of zyxin expression, thereby identifying the miR-16-5p/zyxin axis as a viable therapeutic target for combating intimal hyperplasia in vein grafts (Fig. S4).

SMα-actin and SM22α are specific markers for SMC differentiation, whereas SMC dedifferentiation, proliferation, and migration are marked by OPN and PCNA [17, 33–35]. When triggered by environmental cues such as vein injury resulting from surgical trauma, ischemia-reperfusion injury, and arterialization stress during CABG, venous SMCs can transform from a silent differentiation state to a highly proliferative and migratory dedifferentiated state, resulting in intimal hyperplasia in vein grafts, accompanied by changes in phenotypic marker expression [7–9]. Environmental factors induce SMC phenotypic switching via cytokines such as PDGF-BB, transforming growth factor-β, and insulin growth factor-I. These cytokines are commonly employed in vitro in vascular SMC phenotypic switching models [17, 36–38]. PDGF-BB is the most effective inducer of SMC phenotype switching [39]. Its levels have been found to be higher in vein grafts, where it enhances the proliferation and migration of HSVSMCs [40, 41]. As a result, we employed PDGF-BB to induce SMC phenotypic switching in our investigation.

Although prior research has linked miR-16-5p to BMSC differentiation and cardiomyocyte phenotypic alterations [19, 20], the role of miR-16-5p in venous SMC phenotype switching is unknown. In this investigation, we discovered that miR-16-5p was downregulated in rat vein grafts with intimal hyperplasia and in HSVSMCs exposed to PDGF-BB, which was followed by alterations in phenotypic markers, implying that miR-16-5p reduction is strongly associated with SMC phenotype switching. Significant associations between miR-16-5p expression and phenotypic indicators in PDGF-BB-stimulated HSVSMCs support the role of miR-16-5p downregulation in venous SMC phenotypic switching. It is known that phenotype switching is the initial event in SMC proliferation and migration [42]. Our gain-and loss-of-function tests revealed that miR-16-5p overexpression inhibited PDGF-BB-induced HSVSMC cell proliferation and migration, whereas miR-16-5p silencing promoted it. Several studies have found that miR-16-5p dysregulation is linked to cell proliferation and migration, consistent with our findings. The loss or reduction of miR-16-5p relates to the occurrence of several types of malignancies. Overexpression of miR-16-5p inhibits cancer cell proliferation and migration [43–46]. As a result, our findings show that PDGF-BB induces venous SMC phenotypic switching via downregulation of miR-16-5p, which in turn promotes venous SMC proliferation and migration and, ultimately, intimal hyperplasia.

The miRNAs work biologically by binding to the 3′-UTRs of target genes. However, the miR-16-5p target genes implicated in the phenotypic switching of venous SMCs remain unclear. Our results indicate that miR-16-5p regulates HSVSMCs phenotypic switching by targeting zyxin, as evidenced by base pairing between miR-16-5p and wildtype zyxin-3′-UTR predicted by multiple bioinformatics databases, decreased luciferase activity of the reporter vector containing wildtype zyxin-3′-UTR when co-transfected with miR-16-5p mimics upregulation of zyxin expression. Additionally, restoration of zyxin expression in the presence of miR-16-5p partially but significantly reversed the effect of miR-16-5p overexpression on SMC phenotypic marker expression, indicating that miR-16-5p modulates venous SMC phenotypic switching via zyxin. Given that a single microRNA may target many mRNAs, additional variables may contribute to miR-16-5p signaling in the control of SMC phenotypic switching, which warrants further exploration.

Zyxin is expressed in the extracellular matrix and at the cell-cell junction, where it plays a critical role in stress fiber assembly [47], epithelial-mesenchymal transition (EMT), cell proliferation, migration, and invasion [28]. Zyxin has been shown to stimulate EMT in breast cancer by rearranging microfilaments [48]. In human breast cancer and colorectal cancer, zyxin deficiency reduces cancer cell proliferation and migration [28, 49]. Additionally, zyxin may be used as a phenotypic marker for tumor cells with a high propensity for invasion [50]. These data imply that increased zyxin expression is related to more significant cell proliferation and migration, which is consistent with our findings that zyxin is necessary for HSVSMC cell proliferation and migration.

Mechanical stress induces zyxin expression and subsequent nuclear translocation, where it acts as a mechanotransducer of biological signals [51, 52]. Mechanical stress can also stimulate venous SMCs to switch from a contractile to a synthetic phenotype [53]. Furthermore, stress can stimulate the synthesis and release of PDGF-BB, resulting in phenotypic switching in vascular SMCs [54, 55]. Together with our findings, these data show that environmental stimuli, such as vein injury caused by CABG, trigger the manufacture and release of PDGF-BB, which downregulates miR-16-5p expression, alleviating zyxin suppression, and promoting venous SMC phenotypic switching. Increased expression of miR-16-5p in venous SMCs exposed to external cues may prevent the switch from a contractile to a synthetic phenotype.

## Supplementary Information

Fig. S1 (a) To optimize the delivery of lentiviral vectors into the vein grafts, the external membrane of the vein graft was uniformly coated with 100 μL NOCC/AHA containing 0, 5, 10, or 20 μL (3×10^8^ T.U./mL) Lv-ZsGreen. Green fluorescence was observed under an immunofluorescence microscope. (b) Transduction of Lv-ZsGreen into the brain (left) and contralateral jugular vein (right). (PNG 1285 kb)

High resolution image (TIF 3541 kb)

Fig. S2 MiR-16-5p expression in contralateral jugular veins (PNG 155 kb)

High resolution image (TIF 230 kb)

Fig. S3 Immunofluorescence staining was performed to detect SMα-actin and SM22α expression in isolated HSVSMCs. (a and d) SMα-actin and SM22α were stained in red and green, respectively. (b and e) Nuclei were counterstained with DAPI. (c) (a) and (b) in merge. (f) (d) and (e) in merge. Magnification 200×. (PNG 1383 kb)

High resolution image (TIF 3339 kb)

Fig. S4 A schematic diagram of the miR-16-5p/zyxin axis in the modulation of phenotypic switching of venous SMCs miR-16-5p downregulation mediates venous SMC phenotypic switching, leading to intimal hyperplasia in the vein grafts. miR-16-5p overexpression inhibits the phenotypic transformation of SMCs by repressing zyxin expression, which in turn inhibits intimal hyperplasia. (PNG 768 kb)

High resolution image (TIF 1200 kb)

ESM 1 (DOCX 18 kb)

## Abbreviations

CABG: Coronary artery bypass grafting
SMCs: Smooth muscle cells
CAD: Coronary artery disease
PDGF-BB: Platelet-derived growth factor-BB
SM22α: Smooth muscle 22α
AHA: Aldehyde hyaluronic acid
PBS: Phosphate-buffered saline
OPN: Osteopontin
PCNA: Proliferating cellular nuclear antigen
