# Supplementary figures and images for: miR-16-5p Is a Novel Mediator of Venous Smooth Muscle Phenotypic Switching
Source: J Cardiovasc Transl Res. 2022 May 2;15(4):876–89. doi: 10.1007/s12265-022-10208-1 (PMC9622564; doi:10.1007/s12265-022-10208-1)

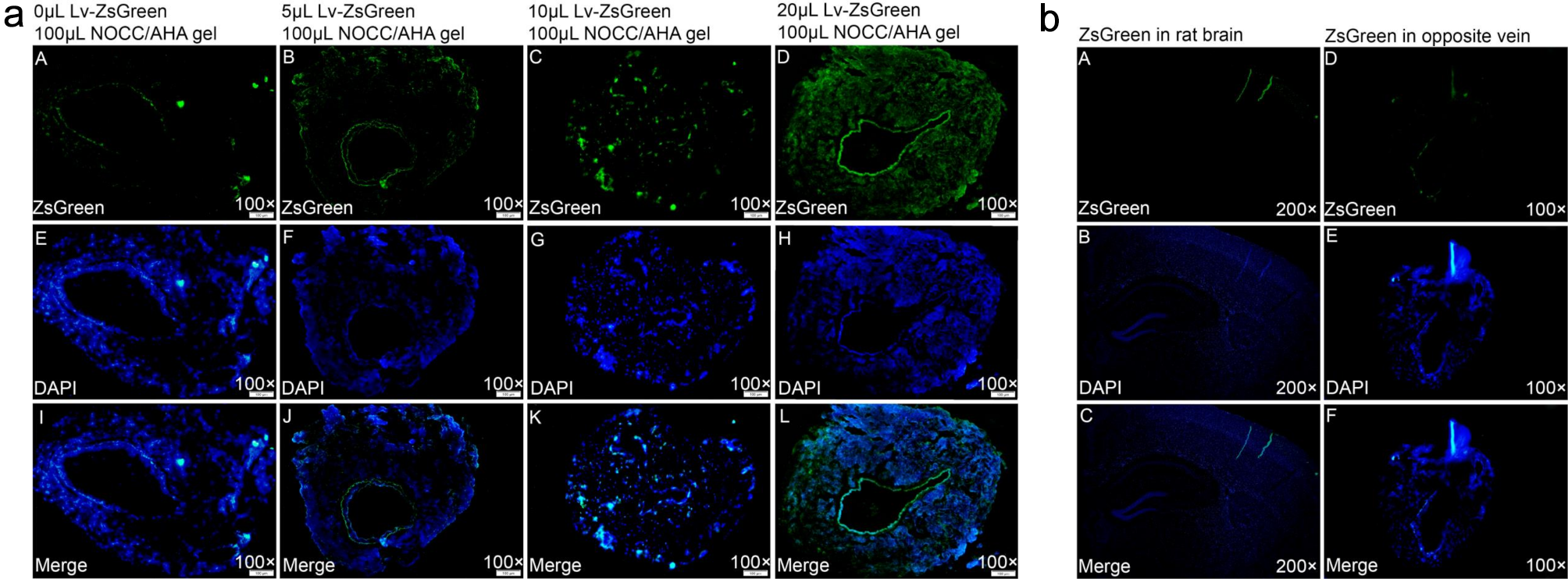

Supplement: Supplementary file 1 — (a) To optimize the delivery of lentiviral vectors into the vein grafts, the external membrane of the vein graft was uniformly coated with 100 μL NOCC/AHA containing 0, 5, 10, or 20 μL (3×10^8^ T.U./mL) Lv-ZsGreen. Green fluorescence was observed under an immunofluorescence microscope. (b) Transduction of Lv-ZsGreen into the brain (left) and contralateral jugular vein (right). (PNG 1285 kb) [file 12265_2022_10208_Fig7_ESM.png]

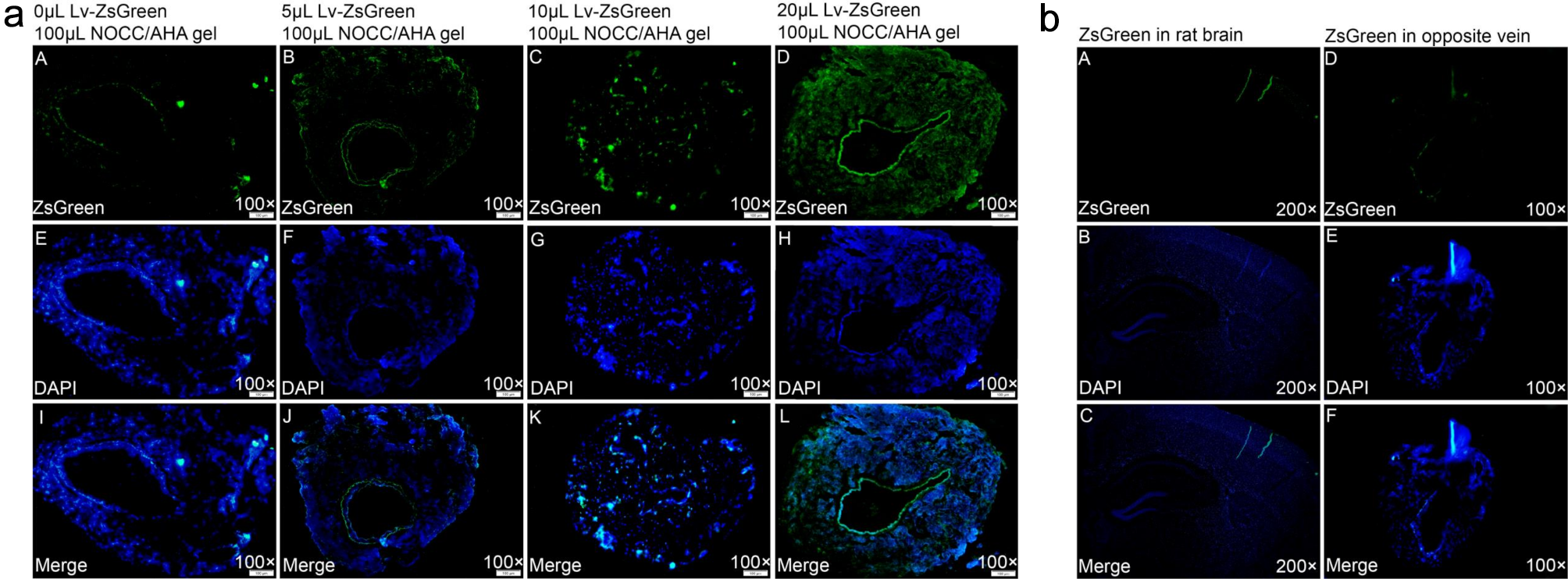

Supplement: Supplementary file 2 — High resolution image (TIF 3541 kb) [file 12265_2022_10208_MOESM1_ESM.tif]

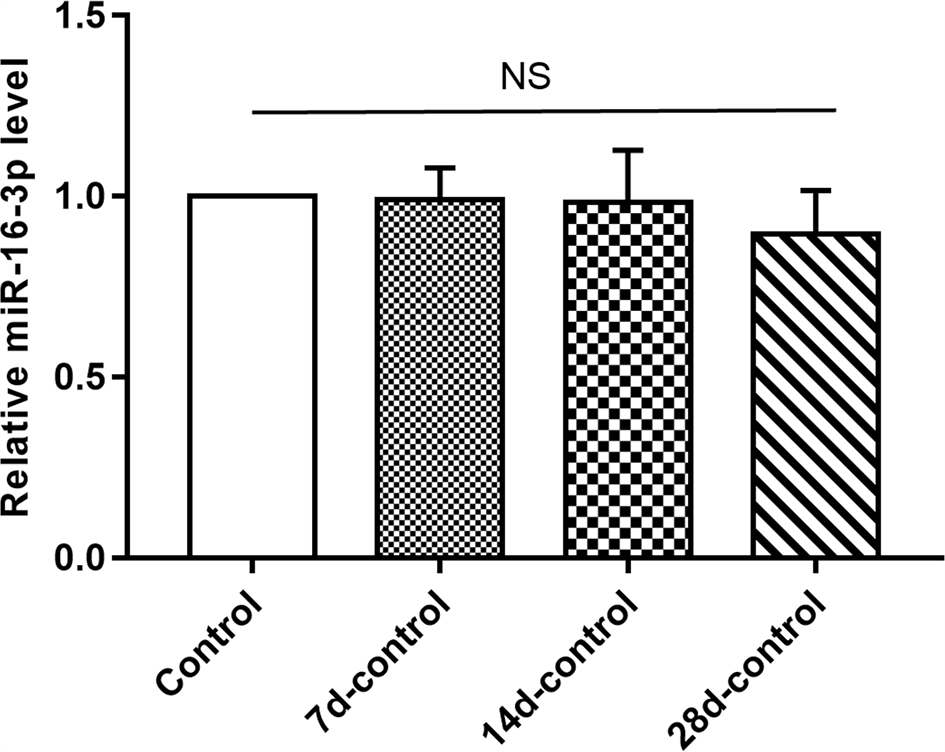

Supplement: Supplementary file 3 — MiR-16-5p expression in contralateral jugular veins (PNG 155 kb) [file 12265_2022_10208_Fig8_ESM.png]

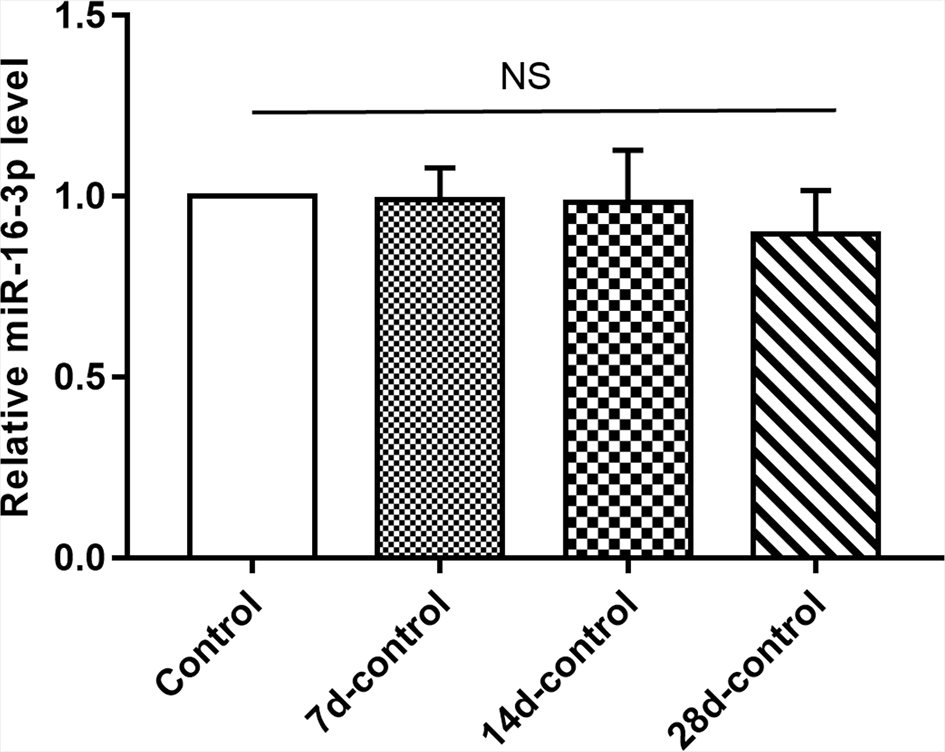

Supplement: Supplementary file 4 — High resolution image (TIF 230 kb) [file 12265_2022_10208_MOESM2_ESM.tif]

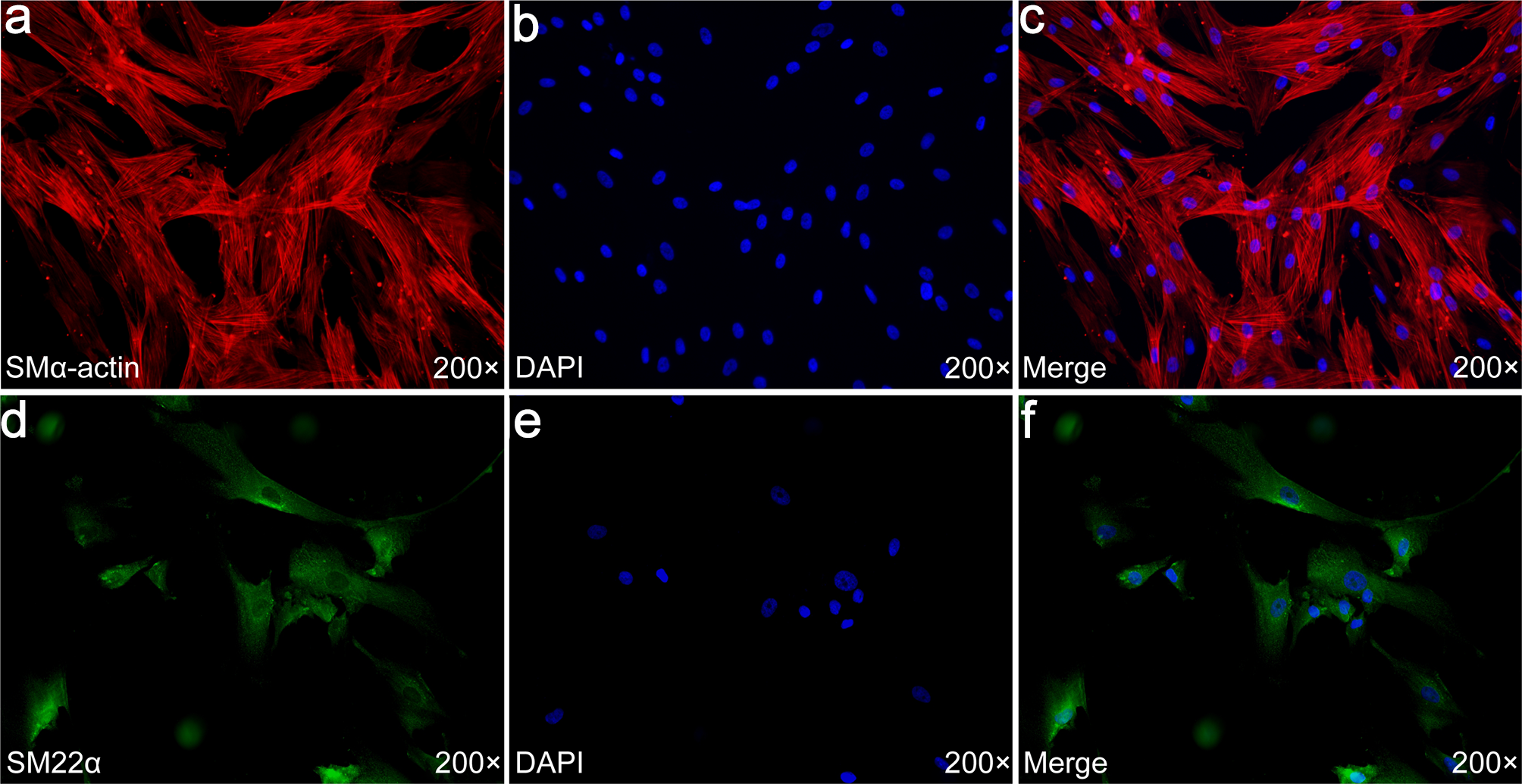

Supplement: Supplementary file 5 — Immunofluorescence staining was performed to detect SMα-actin and SM22α expression in isolated HSVSMCs. (a and d) SMα-actin and SM22α were stained in red and green, respectively. (b and e) Nuclei were counterstained with DAPI. (c) (a) and (b) in merge. (f) (d) and (e) in merge. Magnification 200×. (PNG 1383 kb) [file 12265_2022_10208_Fig9_ESM.png]

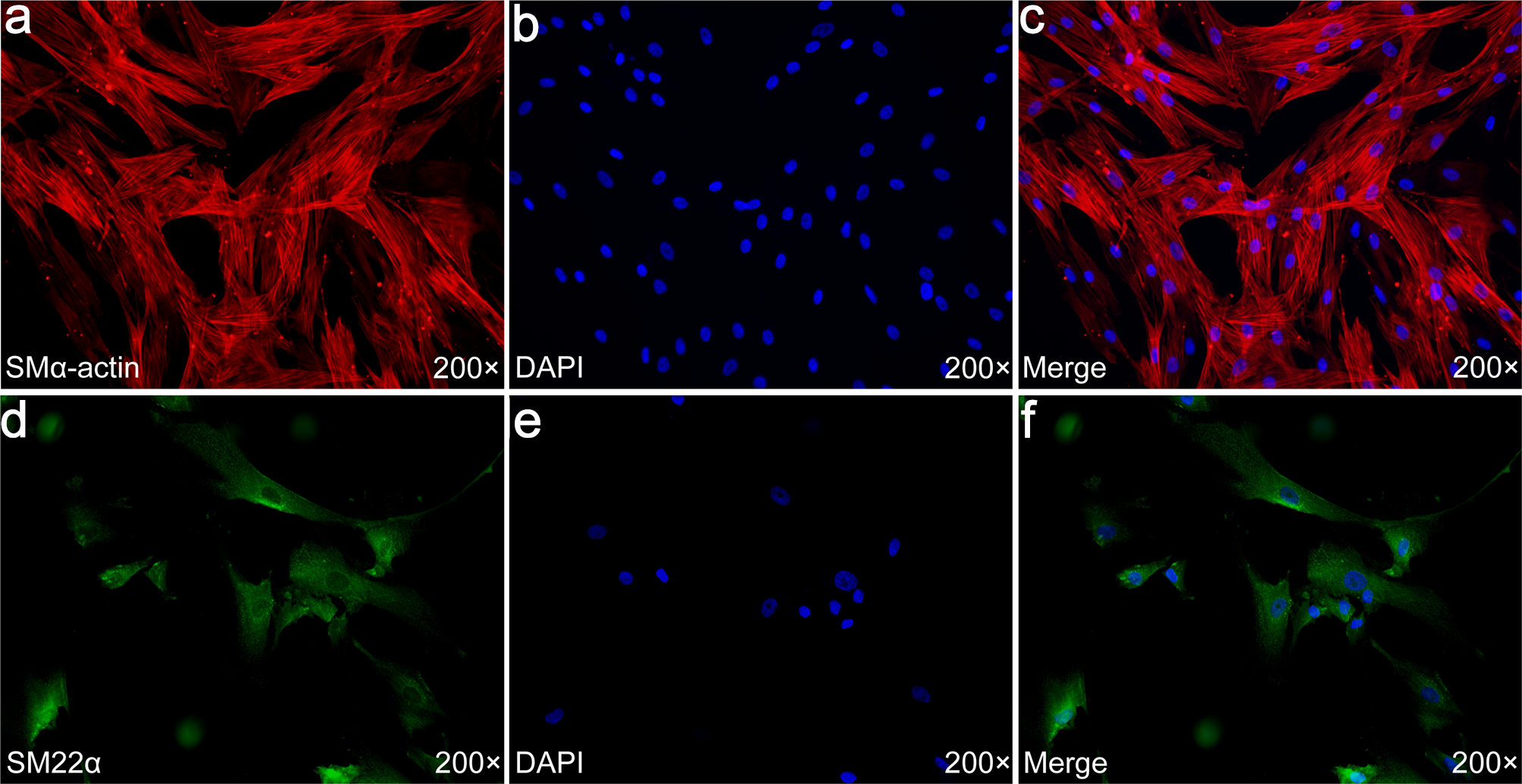

Supplement: Supplementary file 6 — High resolution image (TIF 3339 kb) [file 12265_2022_10208_MOESM3_ESM.tif]

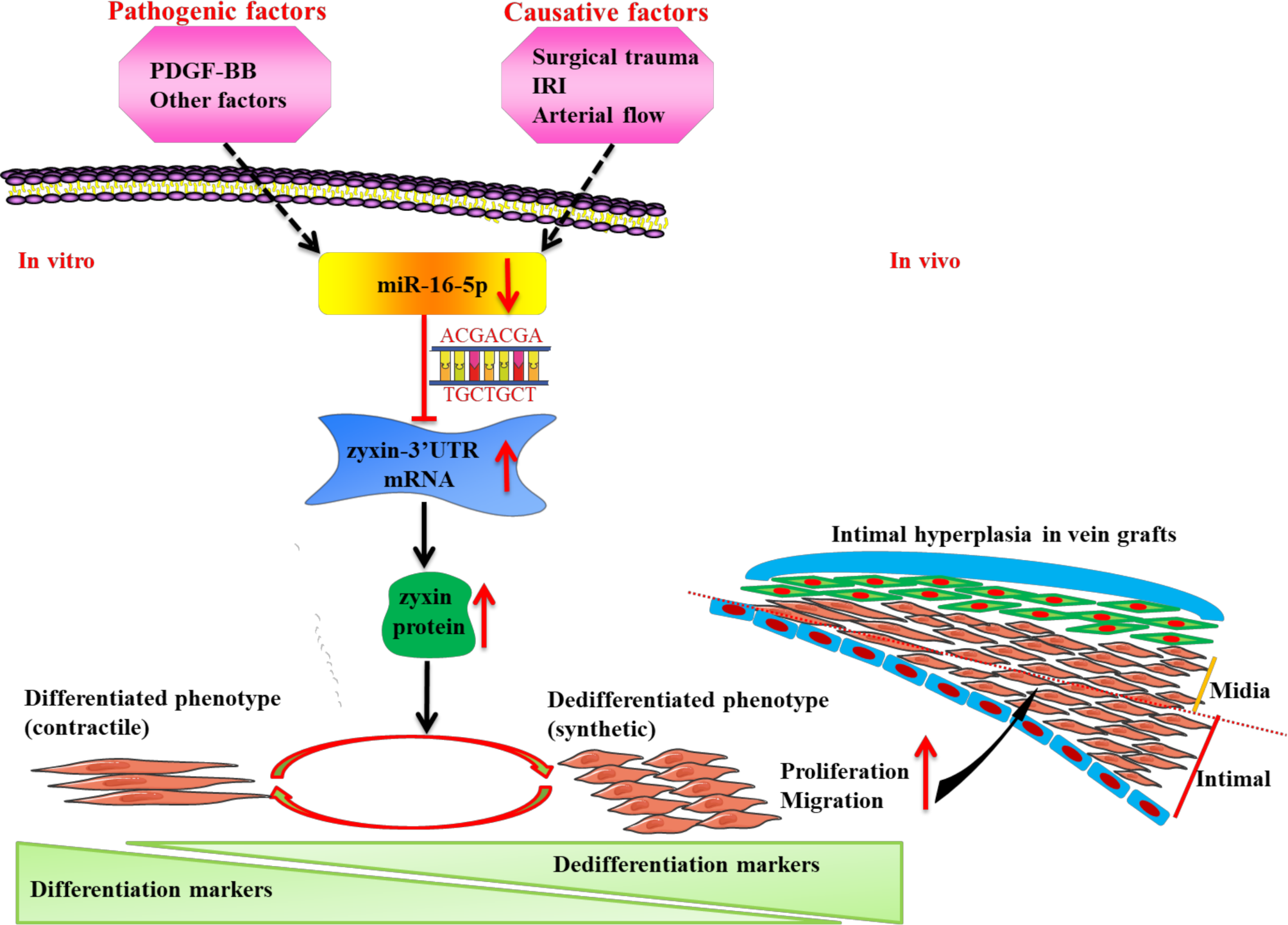

Supplement: Supplementary file 7 — A schematic diagram of the miR-16-5p/zyxin axis in the modulation of phenotypic switching of venous SMCs miR-16-5p downregulation mediates venous SMC phenotypic switching, leading to intimal hyperplasia in the vein grafts. miR-16-5p overexpression inhibits the phenotypic transformation of SMCs by repressing zyxin expression, which in turn inhibits intimal hyperplasia. (PNG 768 kb) [file 12265_2022_10208_Fig10_ESM.png]

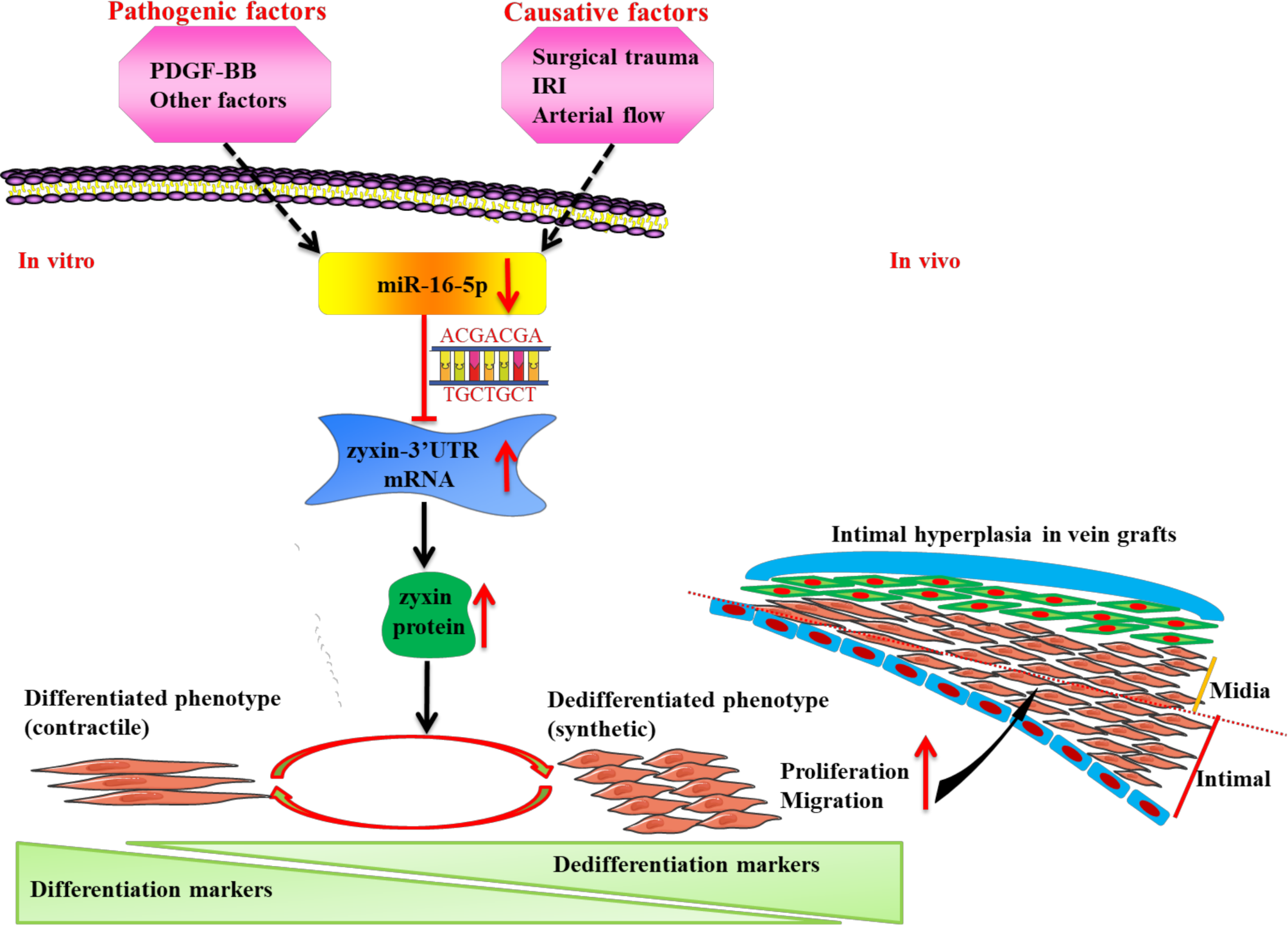

Supplement: Supplementary file 8 — High resolution image (TIF 1200 kb) [file 12265_2022_10208_MOESM4_ESM.tif]
