# Supplementary material for: miR-16-5p Is a Novel Mediator of Venous Smooth Muscle Phenotypic Switching
Source: J Cardiovasc Transl Res. 2022 May 2;15(4):876–89. doi: 10.1007/s12265-022-10208-1 (PMC9622564; doi:10.1007/s12265-022-10208-1)
Supplement: Supplementary file 9 — (DOCX 18 kb) [file 12265_2022_10208_MOESM5_ESM.docx]

**Supplementary Table S1 Primers for quantitative real-time PCR.**

| **Gene/miRNA** | **Forward (5'–3')** | **Reverse (5'–3')** |
| --- | --- | --- |
| miR-16-5p | CGCGTAGCAGCACGTAAATA | AGTGCAGGGTCCGAGGTATT |
| miR-145 | CGGTCCAGTTTTCCCAGGA | AGTGCAGGGTCCGAGGTATT |
| U6 | CTCGCTTCGGCAGCACATATACT | ACGCTTCACGAATTTGCGTGTC |
| Human zyxin | GCAGAATGTGGCTGTCAACGAAC | TGAAGCAGGCGATGTGGAACAG |
| Human SMα-actin | TCCCTTGAGAAGAGTTACGAGTT | CATGATGCTGTTGTAGGTGGTT |
| Human SM22α | TCCAGACTGTTGACCTCTTTG | TCTTATGCTCCTGCGCTTTC |
| Human OPN | TGATGCTACAGACGAGGAC | ACTATCAATCACATCGGAAT |
| Human PCNA | AACCTGCAGGCATGGACTC | TCATTGCCGGCGCATTTTAG |
| Human GAPDH | CACCCACTCCTCCTACTTT | CCCTGTTGCTGTAGCCAAAT |
| Rat SMα-actin | GTCAGGTCATCACTATCGGCAAT | GAAGGAATAGCCACGCTCAG |
| Rat SM22α | TGGCTGAAGAATGGCGTGAT | TCCATCGTTCTTGGTCACGG |
| Rat OPN | TGATGACGACGACGATGACGATGG | ACGCTGGGCAACTGGGATGACCTT |
| Rat PNCA | ACTTGGAATCCCAGAACAGG | CACAGCATCTCCAATATGGC |
| Rat Zyxin | GGCTGCTACACCGACACTTTG | CTCAGCATGCGGTCAGTGAT |
| Rat GAPDH | GGTGATGCTGGTGCTGAGTAT | AGATGATGACCCTTTTGGCCCC |
